# Validating and enabling phosphoglycerate dehydrogenase (PHGDH) as a target for fragment-based drug discovery in PHGDH-amplified breast cancer

**DOI:** 10.18632/oncotarget.11487

**Published:** 2016-08-22

**Authors:** Judith E. Unterlass, Arnaud Baslé, Timothy J. Blackburn, Julie Tucker, Céline Cano, Martin E.M. Noble, Nicola J. Curtin

**Affiliations:** ^1^ Northern Institute for Cancer Research, Medical School, Newcastle University, Newcastle upon Tyne, NE2 4HH, UK; ^2^ Institute of Cell and Molecular Biosciences, University of Newcastle, Newcastle upon Tyne, NE2 4HH, UK; ^3^ Northern Institute for Cancer Research, School of Chemistry, Newcastle University, Newcastle upon Tyne, NE1 7RU, UK

**Keywords:** cancer metabolism, PHGDH, serine metabolism, drug discovery, fragments

## Abstract

3-Phosphoglycerate dehydrogenase (PHGDH) has recently been identified as an attractive target in cancer therapy as it links upregulated glycolytic flux to increased biomass production in cancer cells. PHGDH catalyses the first step in the serine synthesis pathway and thus diverts glycolytic flux into serine synthesis. We have used siRNA-mediated suppression of PHGDH expression to show that PHGDH is a potential therapeutic target in *PHGDH*-amplified breast cancer. Knockdown caused reduced proliferation in the *PHGDH*-amplified cell line MDA-MB-468, whereas breast cancer cells with low PHGDH expression or with elevated PHGDH expression in the absence of genomic amplification were not affected. As a first step towards design of a chemical probe for PHGDH, we report a fragment-based drug discovery approach for the identification of PHGDH inhibitors. We designed a truncated PHGDH construct that gave crystals which diffracted to high resolution, and could be used for fragment soaking. 15 fragments stabilising PHGDH were identified using a thermal shift assay and validated by X-ray crystallography and ITC competition experiments to exhibit 1.5-26.2 mM affinity for PHGDH. A structure-guided fragment growing approach was applied to the PHGDH binders from the initial screen, yielding greater understanding of the binding site and suggesting routes to achieve higher affinity NAD-competitive inhibitors.

## INTRODUCTION

Deregulated metabolism in cancer provides a variety of potential targets for drug discovery that have yet to be explored. Many tumour types have been shown to preferentially metabolise glucose to lactate, irrespective of oxygen levels, a phenomenon termed the Warburg effect. This aerobic glycolysis process is less efficient in the production of ATP than is oxidative phosphorylation, but provides cancer cells with the means of balancing energy and biomass production [1]. Human 3-phosphoglycerate dehydrogenase (PHGDH) is responsible for the NAD^+^-dependent conversion of 3-phosphoglycerate (3-PG) to phosphohydroxypyruvate (PHP), which is the first step in the *de novo* serine synthesis pathway. Thus, PHGDH activity diverts glycolytic flux into biomass production. Early research showing increased PHGDH activity in rat hepatomas and colon carcinoma compared to matched healthy tissue pointed towards the importance of PHGDH activity in cancer [2, 3]. More recent findings have extended these observations to reveal that *PHGDH*, the gene encoding PHGDH, is frequently amplified in melanoma and certain forms of breast cancer [4, 5]. A previous study had already shown a correlation between high PHGDH-mRNA levels and poor five-year survival in ER-negative basal breast cancers [6]. Consistent with the genetic data, knockdown of PHGDH in melanoma and breast cancer cell lines containing a *PHGDH* amplification, resulted in decreased cell viability [4, 5]. In addition, PHGDH suppression inhibited the growth of mammary tumours in mice, although the suppressive effect seemed to depend on the tumour stage, as a similar *in vivo* study in more established mammary tumours reported no significant reduction in tumour growth following PHGDH knockdown [4, 7]. Another study revealed high PHGDH expression in cervical adenocarcinoma samples, and knockdown of PHGDH in a related xenograft model significantly inhibited cell proliferation and halted tumour progression *in vivo* [8]. Interestingly, overexpression of PHGDH in a non-tumourigenic cell line drove phenotypic alterations typical of malignant transformation [4]. Analysis of PHGDH levels in different cancer cell lines revealed upregulation of PHGDH mRNA rather than changes in enzymatic activity to be the reason for elevated PHGDH activity in human tumour cells [9]. Raised PHGDH mRNA levels have been reported in the colon adenocarcinoma cell line COLO320DM and in the murine lymphoma cell line BW5147.G.1.4 [9]. Subsequent studies showed significantly enhanced PHGDH expression in melanoma and breast cancer, indicating that these cancer types might be susceptible to treatment by PHGDH inhibition [4-6]. In addition, PHGDH expression was found to correlate with tumour grade in glioma cells and tumour stage in cervical cancer [10, 11].

The initial target validation, performed in *in vitro* and *in vivo* knockdown models, points towards PHGDH as a promising target in cancer. However, to date only two studies have reported small molecule inhibitors of PHGDH [12, 13]. The two reported lead compounds CBR-5884 (IC50 = 33 ± 12 μM) [13] and NCT-503 (IC50 = 2.5 ± 0.6 μM) [12] (Figure 3c) have similar potency in *in vitro* biochemical assays. Both compounds were shown to inhibit PHGDH activity in a non-competitive manner and to selectively target cancer cell lines dependent on *de novo* serine synthesis [12, 13].

**Figure 1:**
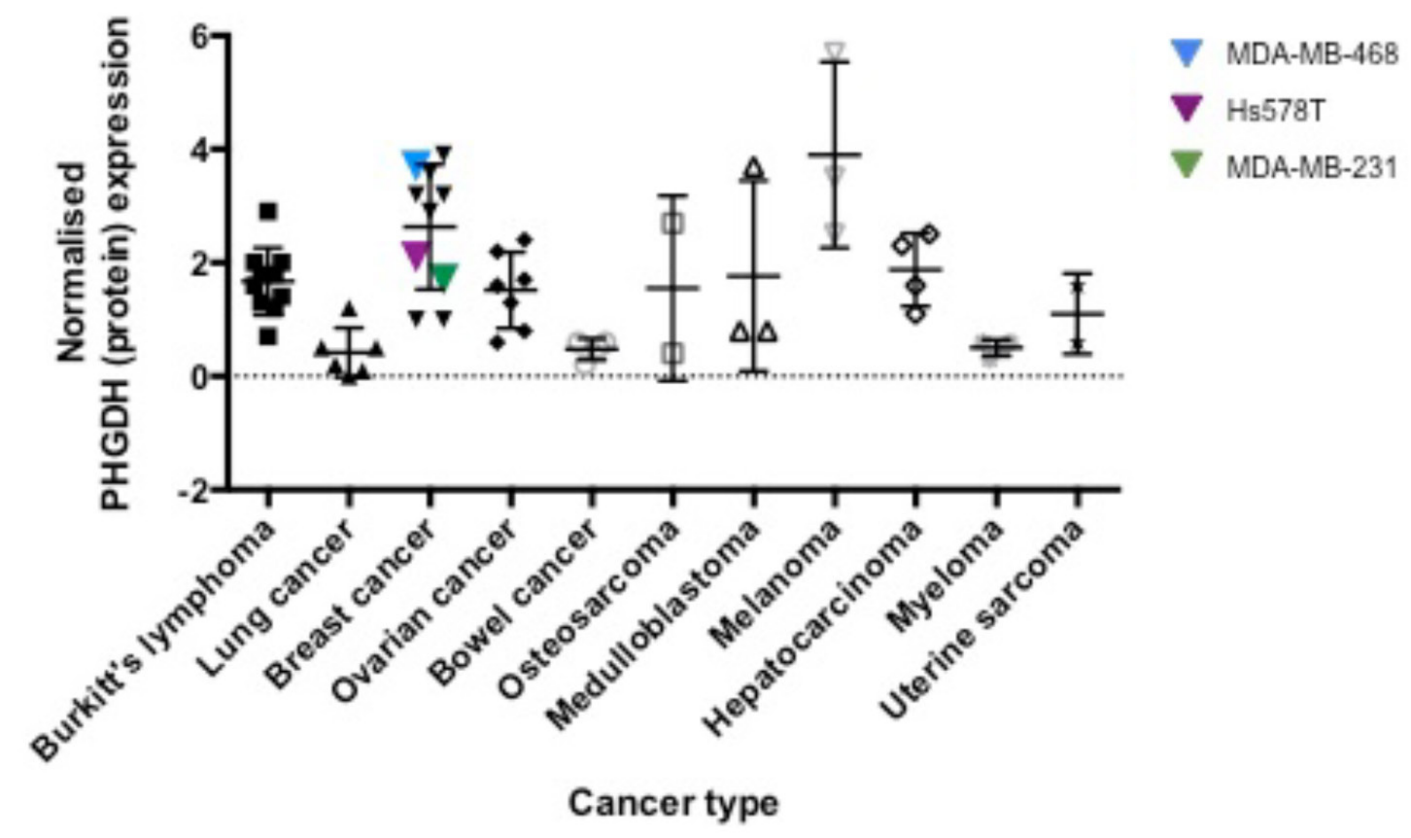
PHGDH protein expression levels in different cancer cell lines PHGDH levels, determined by Western blotting and normalised to total protein levels per sample as determined by Ponceau S stain, as well as the PHGDH protein level in K562 cells as reference point, were grouped according to their tissue of origin.

**Figure 2:**
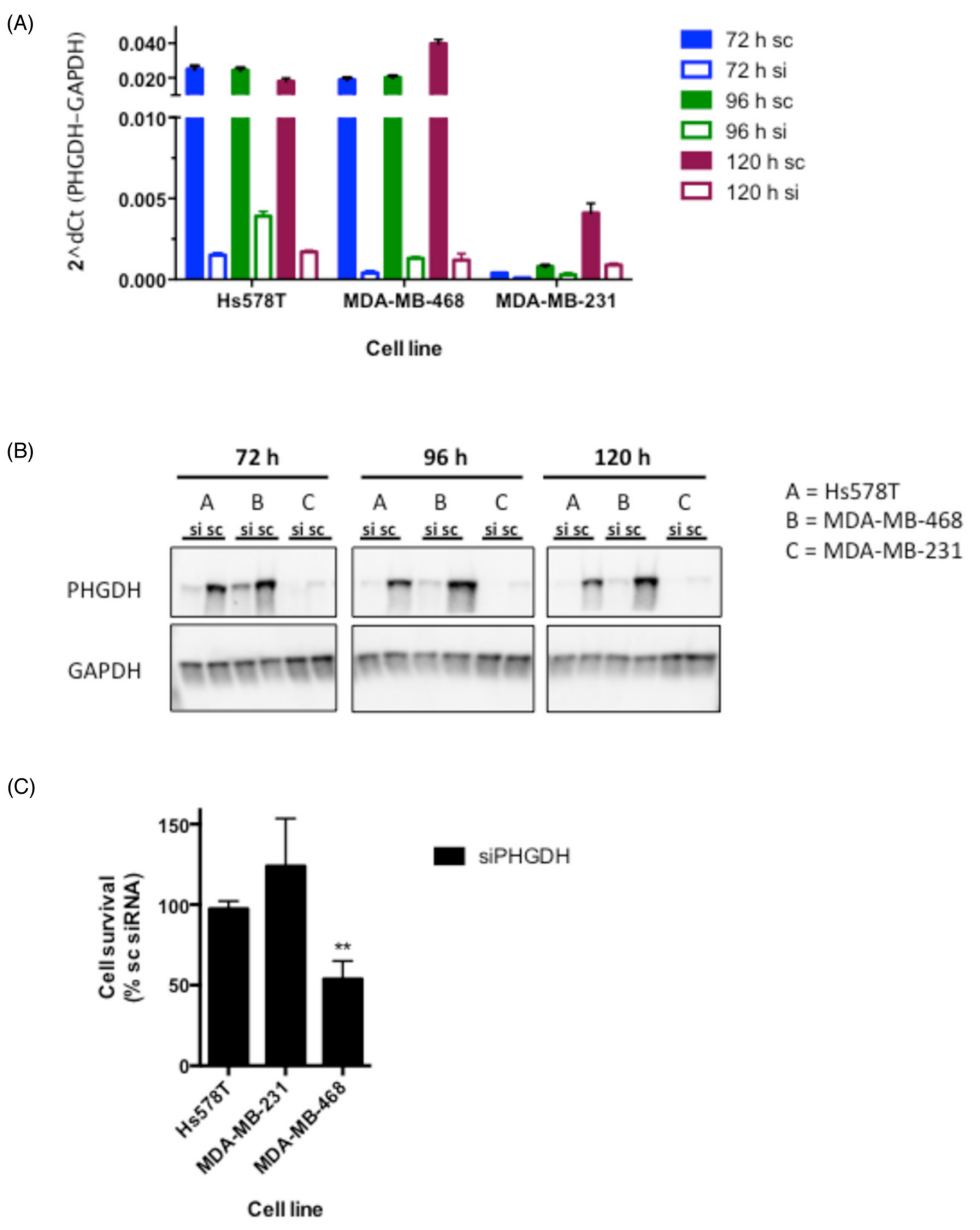
siRNA-mediated knockdown reduces cell proliferation in MDA-MB-468 breast cancer cells **A.** PHGDH mRNA levels following 72-120 h siRNA-mediated knockdown in three breast cancer cell lines. mRNA expression of PHGDH was normalised to GAPDH levels. **B.** Western blot analysis of PHGDH levels in breast cancer cell lines following PHGDH knockdown. **C.** Cell proliferation measurements determined by colony formation assays. Graph represents averages and standard deviations of three independent experiments with three intra-assay repeats. Data is normalised to the respective scrambled siRNA control.

**Figure 3:**
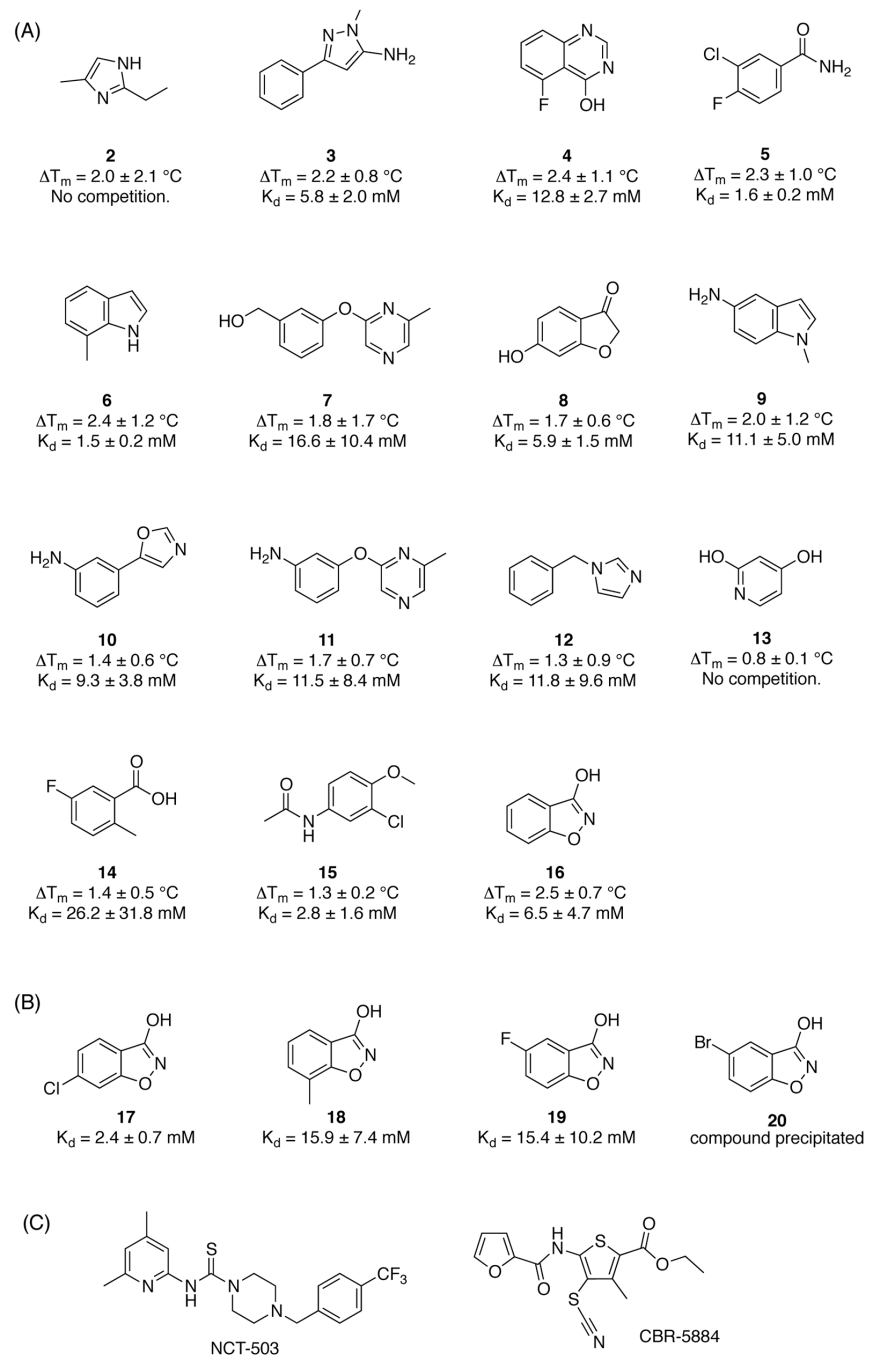
Screening hits and elaborated hit molecules for PHGDH **A.** Structures are presented for the subset of hits identified by primary DSF screen that were further evaluated in an ITC competition experiment. **B.** Modified fragments from the initial screening that were subsequently evaluated in an ITC competition experiment. **C.** Structures of reported PHGDH inhibitors CBR-5884 and NCT-503.

In an effort to further build on the reported target validation and to provide a chemical probe to interrogate the role of PHGDH activity in cancer, we explored the effects of PHGDH knockdown in a panel of cancer cell lines, and developed assays and a crystal system for the identification of fragments that bind to the cofactor binding-site of PHGDH.

## RESULTS

### PHGDH expression in different cancer types

In order to extend the reported analyses into other cancer types, we analysed PHGDH protein levels by Western blot in a panel of 50 different cell lines belonging to ten different cancer types (Figure 1, Supplementary Figure S1). The observed PHGDH expression levels varied from almost no expression to 6-fold higher expression compared to K562 cells. Of the cell lines investigated, those derived from breast cancer and melanoma demonstrated the highest levels of PHGDH expression (Figure 1). The observed higher expression of PHGDH in breast cancer and melanoma cells is in line with previous observations regarding elevation of PHGDH in these cell types and their potential dependence on PHGDH-mediated supply of oncometabolites [4, 5, 14, 15].

### *PHGDH*-overexpressing MDA-MB-468 are susceptible to PHGDH knockdown

Based on our PHGDH protein expression analysis together with previous findings reporting PHGDH overexpression in a subset of ER^-^ breast cancer cell lines, we chose to further assess the effect of PHGDH knockdown in three different breast cancer cell lines [4, 7]: two that over-express PHGDH (MDA-MB-468, Hs578T) and one that expresses relatively little PHGDH (MDA-MB-231). siRNA-mediated knockdown by a pool of four sequence-independent siRNA oligonucleotides resulted in significantly reduced PHGDH mRNA and protein levels over at least 120 hours in all three cell lines (Figure 2A and 2B). Following successful knockdown, cell viability was determined in colony formation assays. A significant decrease in viability was seen in MDA-MB-468 cells, whereas the clonogenic potential of Hs578T and MDA-MB-231 cells was not affected (Figure 2C). The reduced viability of MDA-MB-468 cells following PHGDH knockdown supports previous findings from other groups [4, 7], however, no effect of PHGDH knockdown was seen in Hs578T cells despite similar PHGDH expression in both cell lines.

### Differential Scanning Fluorimetry (DSF) screening of a fragment library reveals moderate affinity binders to PHGDH

In order to identify new PHGDH binders structurally different from CBR-5884 and NCT-503 (Figure 3C), a fragment-based approach was sought. Compared to high throughput screening (HTS), fragment-based drug design (FBDD) allows sampling of a relatively large chemical space with a comparably low amount of compounds. As fragments are of small size and low complexity, the likelihood of binding to the target is increased and the proportion of atoms involved in target binding is higher compared to bigger molecules [16]. Due to the fragments’ low molecular weight (< 300 Da), there is still scope to develop the fragments into more potent molecules with favourable physicochemical properties while staying within the margins of size for a drug-like molecule [17].

In this study a ‘rule-of-three’ [18] compliant library of 600 fragments (CRT Cambridge, UK) was screened against recombinant full-length PHGDH using a differential scanning fluorimetry (DSF) assay. PHGDH has an average melting temperature (T_m_) of 54 °C. Fragments were screened at 1 mM in the presence of 1 % DMSO. 1 mM NADH was used as positive control, and gave an increased T_m_ of 61 °C, corresponding to a Δ T_m_ of 7 °C (Supplementary Figure S2). Hits were determined based on an average Z-score ≥ 1, *i.e*. to a change in T_m_ of PHGDH equal to or greater than one standard deviation of the mean (Supplementary Table S1). Applying this threshold, 42 compounds were classified as hits, which corresponds to a hit rate of 6.4 %. A subset of 15 fragments was commercially available and were repurchased and further evaluated in an ITC competition experiment (Figure 3, Table 1, Supplementary Figure S3). The ITC competition experiments confirmed binding of 13 fragments to the cofactor-binding pocket of PHGDH. Two fragments did not affect the binding of NADH to PHGDH in this experimental setup, which suggests that these fragments are unlikely to bind to the cofactor-binding pocket, but does not exclude that they bind at a different site on the protein. In order to further characterise the hits, and to help us prioritise their use as seeds for further hit-expansion studies, we undertook a crystallographic investigation of their respective binding modes.

**Table 1:** Thermodynamic binding parameters determined by ITC competition.

Fragment	ΔH (kJ/mol)	ΔS (kJ/ (mol x K)	ΔG (kJ/mol)	K_d_ (mM)	LE (kJ/(mol x #))
**2**	No competition				
**3**	-467.2	-1.61	12.8	5.8 ± 2.0	0.23
**4**	8.3	-0.01	10.8	12.8 ± 2.7	0.21
**5**	-5.4	-0.07	16.0	1.6 ± 0.2	0.35
**6**	-1593.9	-5.40	16.1	1.5 ± 0.2	0.38
**7**	15.8	0.02	10.2	16.6 ± 10.4	0.15
**8**	-292.7	-1.02	12.7	5.9 ± 1.5	0.28
**9**	-102.0	-0.38	11.2	11.1 ± 5.0	0.24
**10**	-260.8	-0.91	11.6	9.3 ± 3.8	0.23
**11**	-11.5	-0.08	11.1	11.5 ± 8.4	0.18
**12**	-64.4	-0.25	11.0	11.8 ± 9.6	0.22
**13**	No competition				
**14**	-47.0	-0.19	9.0	26.2 ± 31.8	0.20
**15**	-1753.2	-5.93	14.5	2.8 ± 1.6	0.27
**16**	-382.6	-1.33	12.5	6.5 ± 4.7	0.30
**17**	-1517.3	-5.14	14.9	2.4 ± 0.7	0.32
**18**	-194.3	-0.67	10.3	15.9 ± 7.4	0.22
**19**	-190.1	-0.67	10.3	15.4 ± 10.2	0.22
**20**	Compound precipitated				

Changes in entropy (ΔS) and enthalpy (ΔH) as well as the binding constant (K_D_) were determined. LE was calculated as the coefficient of ΔG per number of non-hydrogen atoms (= #).

### Validation of fragments by X-ray crystallography

A crystal structure of the catalytic subunit of PHGDH (amino acids (aa) 3-314) with cofactor, NAD^+^, and substrate analogue, *L*-malate (1), bound to the active site has been reported (PDB 2G76). Although crystallisation of this catalytic domain of PHGDH construct was reproducible in our hands, the crystals formed intergrown stacks of sheet-like crystals and diffracted to a rather low resolution of 2.7 Å (data not shown). In addition, the active site in the crystals was partially occupied by the natural cofactor, which co-purified with the protein and was difficult to remove subsequently. In order to identify more suitable protein constructs for crystallisation, limited proteolysis was performed. This approach has been shown (reviewed in 14, 15) to be successful in increasing the propensity of proteins to crystallise because proteases primarily cut at solvent accessible and flexible or unstructured sites, thereby reducing the target to relatively stable protein cores [19, 20].

**Figure 4:**
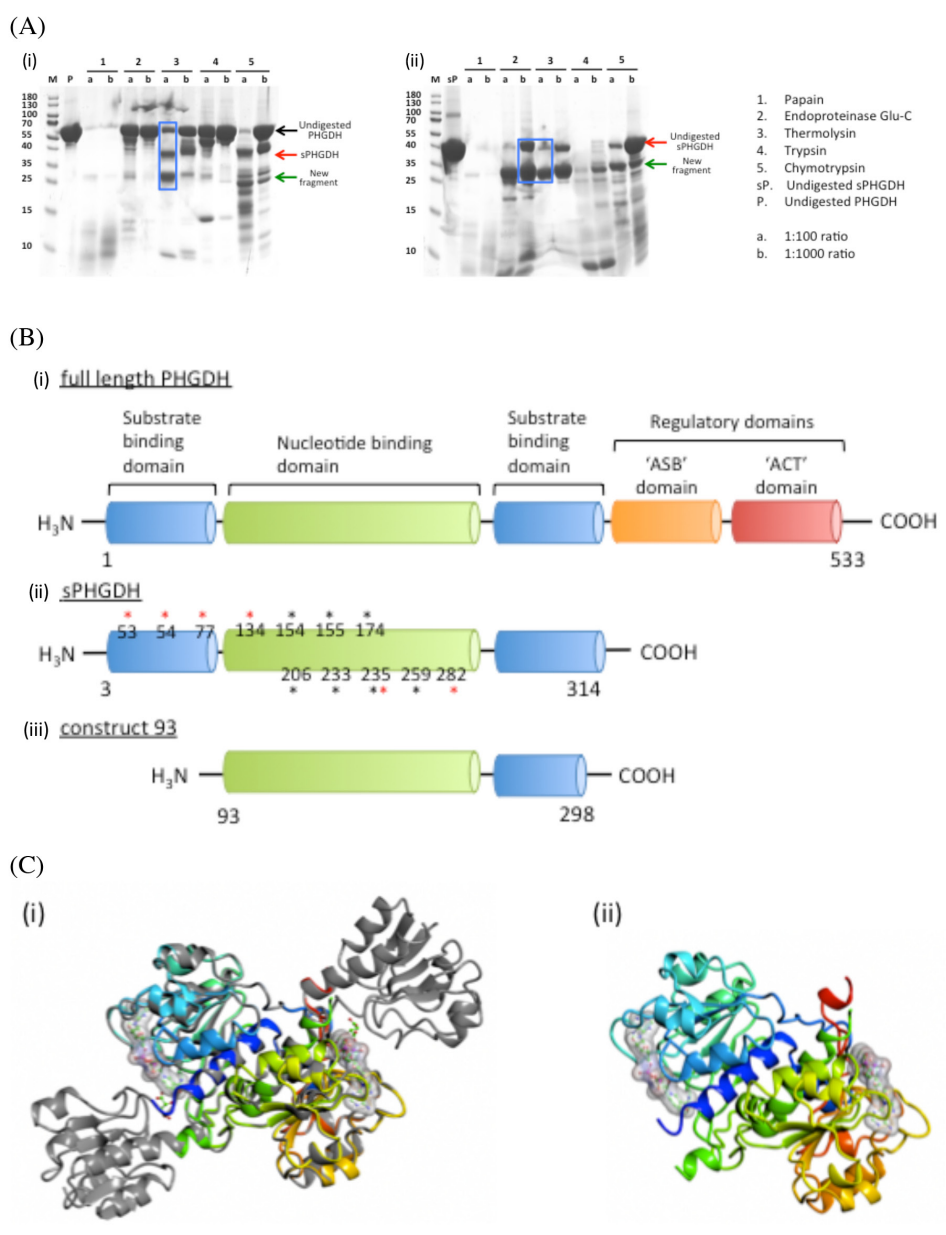
Limited proteolysis and domain composition of PHGDH **A.** 100 µg or 50 µg PHGDH or sPHGDH was mixed with five different proteases at molar ratios of 1:100 and 1:1000. The digestion was performed for 1 h at RT or 37 °C and analysed by SDS-PAGE. **B.** A comparison of the structural elements comprising human PHGDH, sPHGDH and PHGDH-93. Amino acids involved in hydrogen bond formation to the substrate (*) or cofactor NAD^+^ (*) are highlighted. **C.** A comparison of the 3D structures of sPHGDH and PHGDH-93. (i) A ribbon diagram of PHGDH-93 (coloured model) is shown superimposed on sPHGDH (grey). (ii) Ribbon diagram of PHGDH-93 with bound cofactor NAD^+^.

Proteolysis was performed by mixing PHGDH with five different proteases separately (papain, endoproteinaseGlu-C, thermolysin, trypsin and chymotrypsin) in 1:100 and 1:1000 molar ratios. The mixtures were incubated at room temperature (RT) or 37 °C for one hour and analysed by SDS-PAGE (Figure 4). Cleanest cleavage was seen with thermolysin at 37 °C which resulted in cleavage of PHGDH into two fragments, one of 36 kDa, which is likely to correspond to the shorter PHGDH fragment (aa 3-314) already used for crystallisation, and a smaller fragment of 26 kDa (Figure 4, lane 7).

Subjection of the 26 kDa cleavage product to ESI-MS, combined with knowledge of the preferred cleavage sites of thermolysin, allowed us to narrow down the potential amino acid sequences of the proteolytically-derived PHGDH fragments (Supplementary Figure S4). According to this analysis, the most abundant product of proteolysis corresponded to amino acids 93-298. Accordingly, we prepared a His-tagged *E. coli* expression construct for a truncated protein corresponding to this fragment (termed PHGDH-93), which could be recombinantly expressed and purified by affinity and gel filtration chromatography. The measured OD_260/280_ ratio of 0.71 for purified PHGDH-93 indicated a degree of contamination with co-purifying cofactor (NAD^+^ and/or NADH), and therefore the protein was subjected to charcoal treatment prior to crystallisation. PHGDH-93 was then successfully crystallised with an empty, accessible cofactor-binding pocket. Comparison with the available PHGDH structure (2G76) showed that PHGDH-93 was missing a lid domain, which partly encloses the substrate-binding site. As PHGDH-93 does not contain this lid domain necessary for substrate binding, the protein construct lacks catalytic activity compared to sPHGDH (data not shown). However, the core domain of PHGDH, which includes the cofactor-binding site, was properly folded in PHGDH-93 and overlaid well onto the published structure as well as giving stable crystals (Figure 4C). Thus PHGDH-93 provides a valuable tool to investigate binders to the cofactor-binding site.

**Figure 5:**
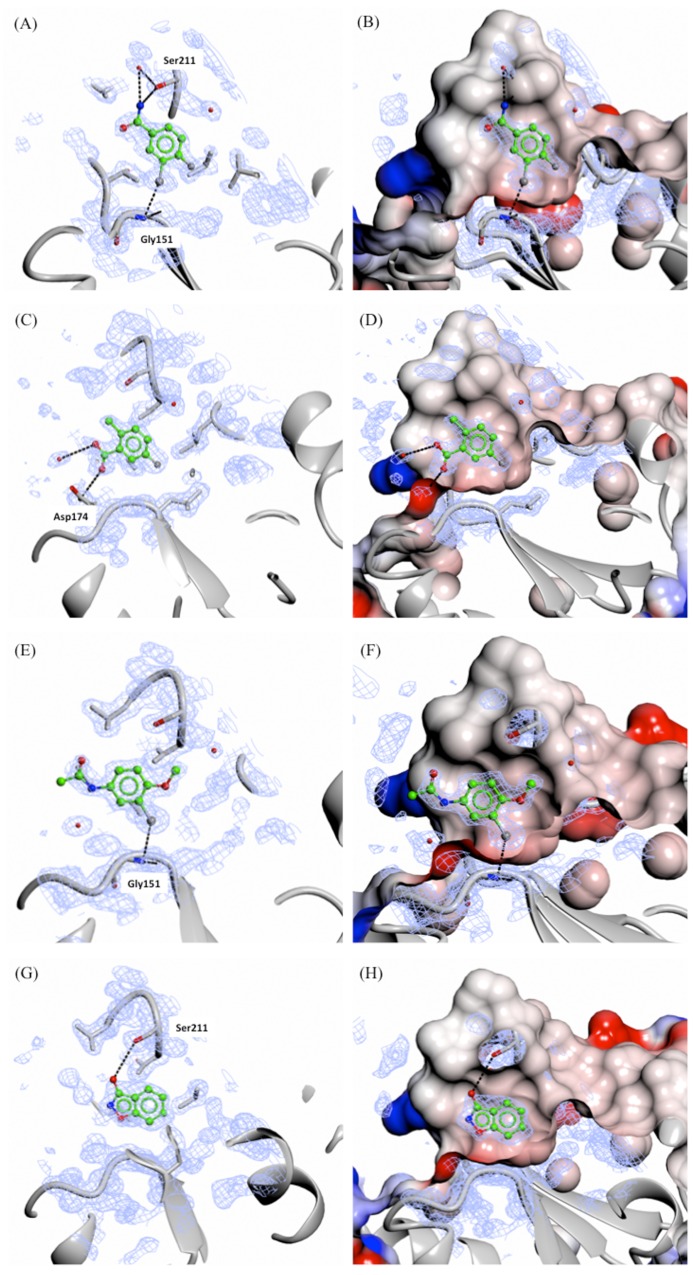
X-ray crystal structures of fragments forming specific hydrogen and/or halogen bonds with PHGDH Crystal structures of bound fragment 5 (**A.** and **B.**, 1.53 Å resolution), 14 (**C.** and **D.**, 1.53 Å resolution), 15 (**E.** and **F.**, 1.53 Å resolution) and 16 (**G.** and **H.**, 1.33 Å resolution) are shown. The F_o_-F_c_ omit electron density maps are shown as green wire at contour levels of 0.5 electrons/Å^3^. The left-hand panels show the protein as a grey ribbon representation with selected side-chains drawn as sticks with carbon atoms in grey. The right-hand panels show the protein surface coloured by electrostatic potential.

**Figure 6:**
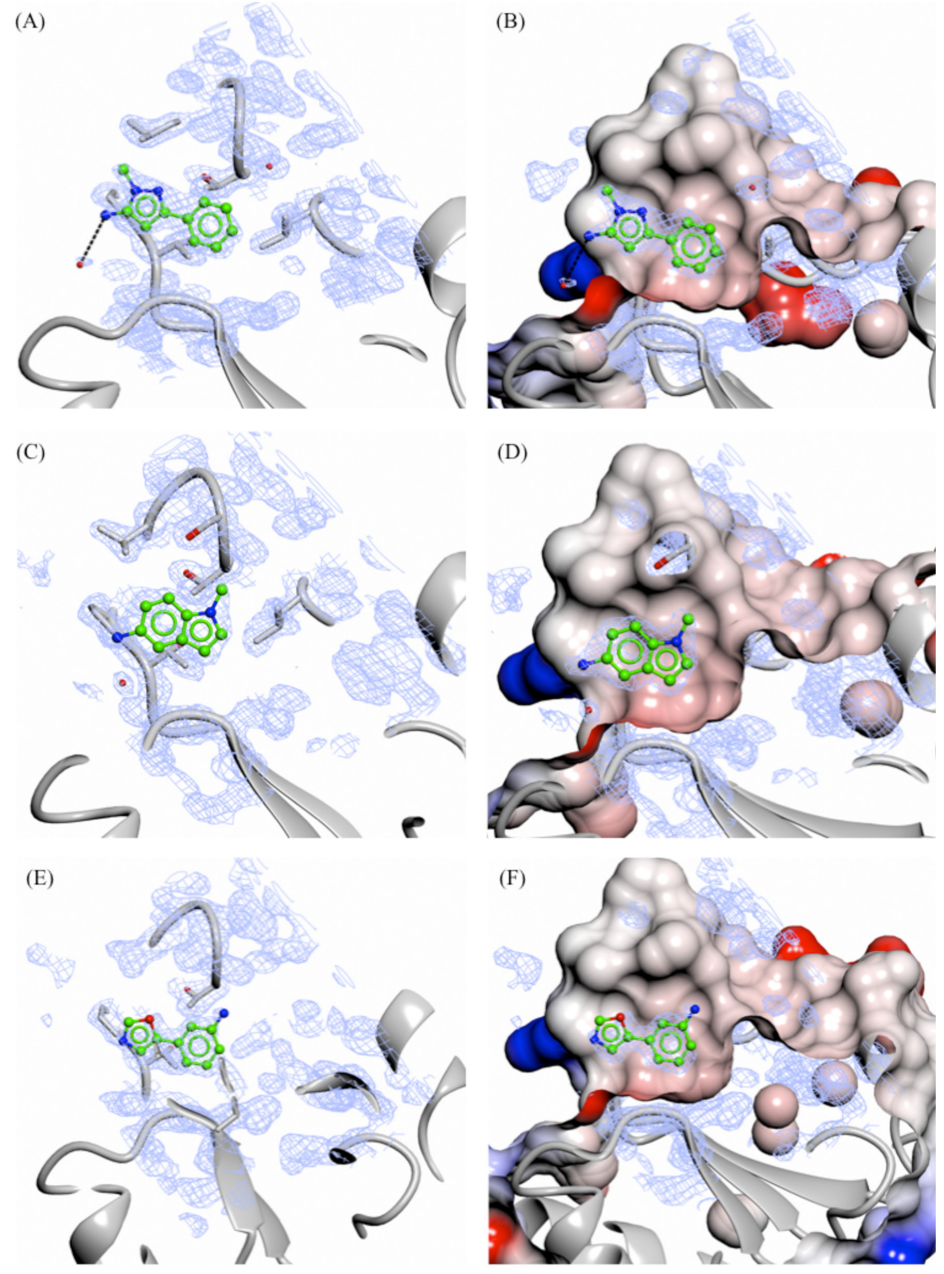
X-ray crystal structures of fragments not showing specific interactions bound to the adenine-subsite of PHGDH Crystal structures of bound fragment 3 (**A.** and **B.**, 1.33 Å resolution), 9 (**C.** and **D.**, 1.53 Å resolution) and 10 (**E.** and **F.**, 1.53 Å resolution) are shown. The F_o_-F_c_ omit electron density map is shown as green wire at contour levels of 0.5 electrons/Å^3^. The left-hand panels show the protein as a grey ribbon representation with selected side-chains drawn as sticks with carbon atoms in grey. The right-hand panels show the protein surface coloured by electrostatic potential.

Soaking of all 15 fragments into PHGDH-93 crystals was performed overnight at RT using a fragment concentration of 80 mM in 20 % DMSO. X-ray diffraction data were collected from the soaked crystals at the DIAMOND Light Source (DLS) to resolutions of between 1.3 and 1.7 Å (Supplementary Table S2). Difference electron density corresponding to seven fragments (3, 5, 9, 10, 14-16, 47 % of tested compounds) was clearly visible. All of the fragments for which crystallographic data were obtained bound to the adenine-pocket suggesting this pocket to be a preferential site for fragment binding (Figures 5 and 6). Analysis of the fragments’ binding modes showed that four of the fragments (5, 14, 15 and 16) were coordinated by hydrogen and/or halogen bonds (Figure 5), whereas the other three fragments (3, 9 and 10) appeared to be stabilised only by apolar contacts (Figure 6). Of the four compounds forming specific interactions with the protein, fragments 5 (3-chloro-4-fluorobenzamide), 14 (5-fluoro-2-methylbenzoic acid) and 15 (*N*-(3-chloro-4-methoxyphenyl)acetamide) constitute substituted benzene rings. The most potent compound according to the ITC data, fragment 5, bound more deeply in the adenine binding pocket compared to the other two structurally similar fragments, and formed an halogen bond with the backbone carbonyl of Gly151 through its chloro atom and a hydrogen bond with the side-chain of Ser211 (Figure 5A and 5B). Fragment 15 formed a similar halogen bond interaction with Gly151 through a chloro-substituent (Figure 5E and 5F). Fragment 14 was substantially less potent (K_d_ = 26 ± 31 mM) compared to the two structurally similar fragments 5 and 15 (5, K_d_ = 1.6 ± 0.2 mM and 15, K_d_ = 2.8 ± 1.6 mM). However, fragment 14 still formed a hydrogen bond with the side-chain of Asp174 and a water molecule present in the binding pocket (Figure 5C and 5D). Fragment 16 (3-hydroxybenzisoxazole) made one hydrogen bond with the side-chain of Ser211 through its hydroxyl functional group (K_d_ = 6.5 ± 5.7 mM) (Figure 5G and 5H). Despite a similar bicyclic structure, fragment 9 (5-amino-1-methyl-1*H*-indole, K_d_ = 11.1 ± 5.0 mM) bound to the adenine pocket in a 180 °-flipped orientation compared to the 3-hydroxybenzisoxazole (16) (Figure 6C and 6D). Thus, the *N*^1^-methylated fragment 9 pointed into a small hydrophobic pocket. The remaining two crystallographically observed fragments, 3 (1-methyl-3-phenyl-1*H*-pyrazol-5-amine) and 10 (3-(1,3-oxazol-5-yl)aniline) were non-fused bicyclic aromatic systems, which, although not forming specific hydrogen bonds to the protein, bound in a similar orientation to one another to the adenine pocket (3, K_d_ = 5.8 ± 2.0 mM and 10, K_d_ = 9.3 ± 3.8 mM) (Figure 6A, 6B, 6E and F).

### Fragment growth allows further exploration of the binding pocket

Based on the fragment crystal structures, fragment 16 was selected for modification to further explore the binding site and potential vectors for fragment growth.

**Figure 7:**
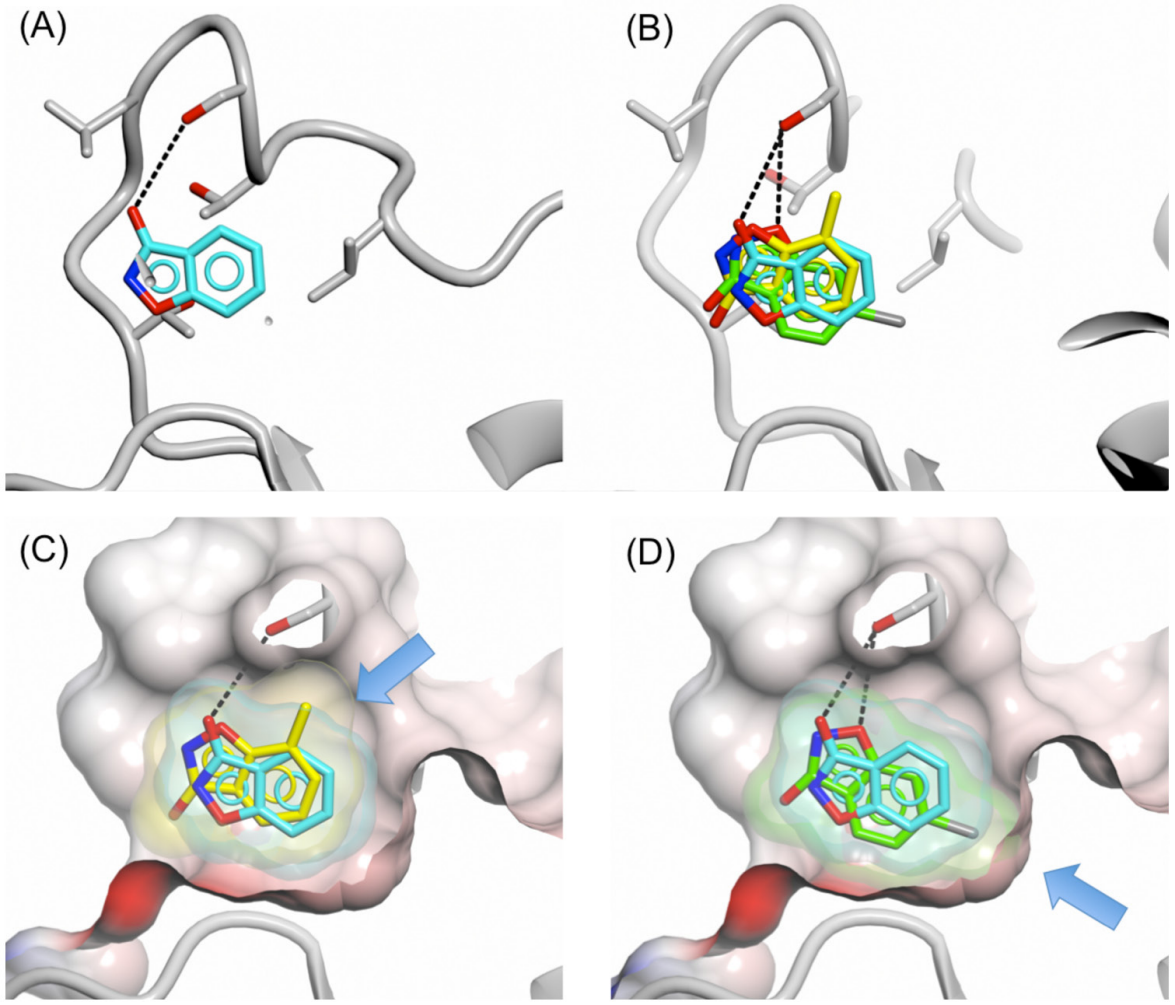
Binding of 3-hydroxybenzisoxazole and analogues with substituents at positions 5 and 7 Binding of fragments 16 (cyan), 17 (yellow) and 18 (green) to the adenine-binding pocket of PHGDH-93. **A.** Fragment 16, **B.** fragments 16, 17 and 18, **C.** fragments 16 and 18, **D.** fragments 16 and 17. The protein is shown in ribbon representation in grey together with the protein surface coloured by electrostatic potential. The compound surfaces are coloured to match the carbon atoms of the respective fragment. Hydrogen bonds are shown as dashed lines. Blue arrows in pictures (C) and (D) highlight the position of the introduced substituents compared to parent fragment 16.

The binding mode of fragment 16 revealed two potential routes for fragment growth: (1) growth out of the cofactor-binding pocket from C-4 or C-5 and (2) fragment growth from the 3-hydroxy position along the cofactor-binding pocket from the adenine site towards the nicotinamide-binding site. In addition a small hydrophobic pocket adjacent to the fragment was detected that could be filled with a small hydrophobic group by substitution at position C-6 or C-7 of the benzisoxazole so as to increase interactions with the protein. To investigate this small pocket, the 6-chloro (17) and 7-methyl (18) substituted 3-hydroxybenzisoxazoles were investigated. Addition of the chloro-substituent improved fragment binding to sPHGDH as shown by competitive ITC (16, K_d_ = 5.8 ± 4.7 mM and 17, K_d_ = 2.4 ± 0.7 mM), whereas the 7-methyl analogue showed 2.5-fold reduced affinity (18, K_d_ = 15.9 ± 7.4 mM) (Table 1). Crystal structures of these fragments in complex with PHGDH-93 showed that the 7-methyl analogue (18) adopted an orientation flipped by 180° along a horizontal axis compared to fragment 16. In this orientation, the 7-methyl group pointed towards a route for fragment growth out of the pocket (Figure 7C). In addition, the previously formed hydrogen bond between Ser211 and the 3-hydroxy group of fragment 16 (Figure 7A) was no longer formed, but instead the oxygen group within the isoxazole moiety of fragment 18 was brought into close proximity of Ser211 for potential hydrogen bond formation. Fragment 17 adopted the same orientation as fragment 18, forming the previously described hydrogen bond between O-1 and Ser211. In this position the 5-chloro-substituent occupied a small hydrophobic pocket (Figure 7D). This additional interaction with the protein is likely to lead to the observed increase in binding affinity of fragment 17 compared to 16 and 18.

**Figure 8:**
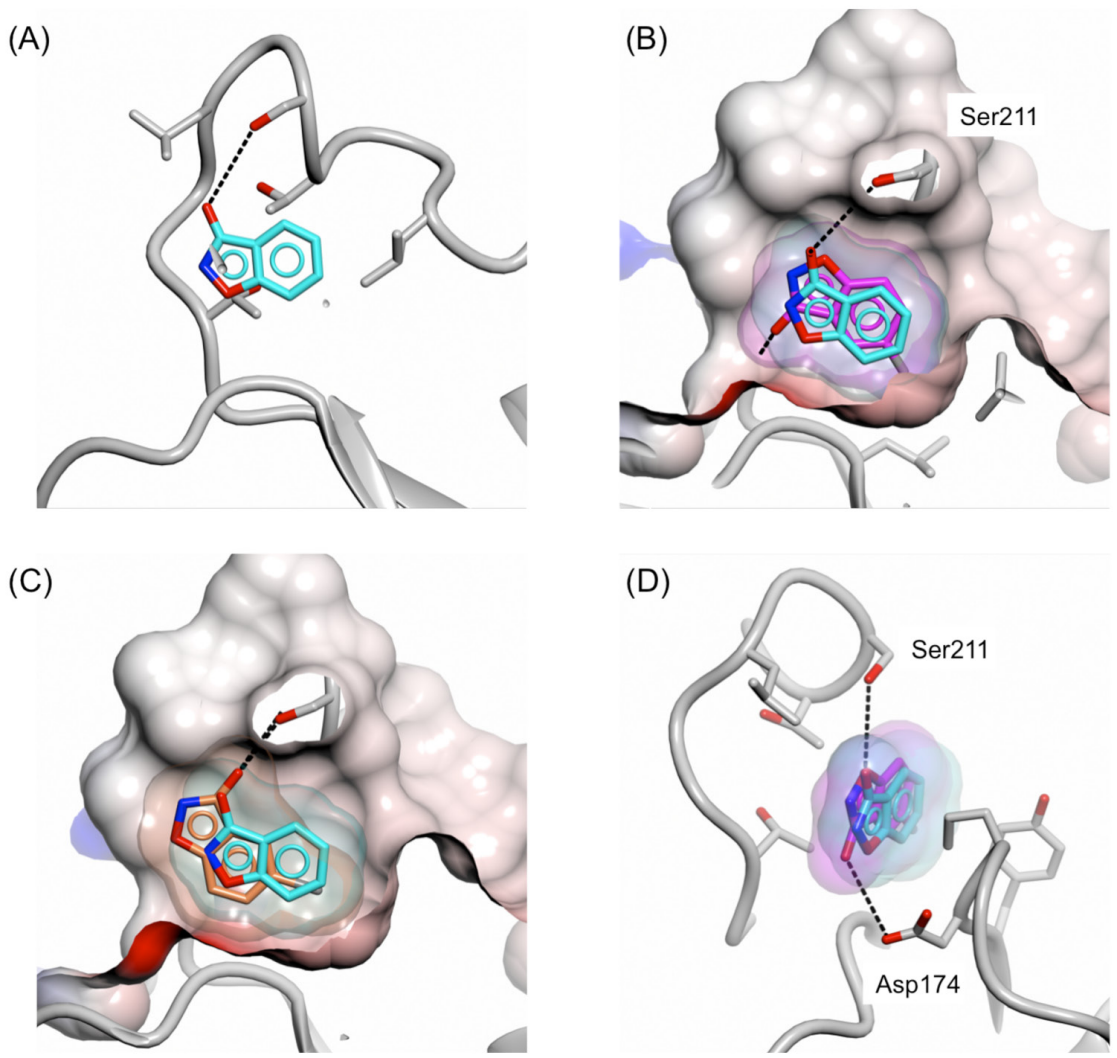
Binding mode of 5-fluoro/bromo-3-hydroxybenzisoxazole to the adenine-binding subsite Binding of fragments 16 (cyan), 19 (magenta) and 20 (coral) to the adenine-binding pocket of PHGDH-93. **A.** Fragment 16, **B.** fragments 16 and 19, **C.** fragments 16 and 20, **D.** fragment 16 and 19. The protein is shown in ribbon representation in grey together with the protein surface coloured by electrostatic potential. The compound surfaces are coloured according to match the carbon atoms of the respective fragment. (D) Hydrogen bonds between fragment 16 or 19 and PHGDH-93 are shown as dashed lines.

The preference of this pocket for small hydrophobic groups was also seen with 3-hydroxybenzisoxazoles carrying halogen substituents at the C-5 position. For example, the crystal structure of PHGDH-93 with 5-fluoro-3-hydroxybenzisoxazole (19) showed that the fluoro-group occupied also the hydrophobic pocket whilst adopting a similar orientation as the 6-chloro analogue (17) (Figure 8B). However, with the fluoro-substituent being in the 5-position, fragment 19 adopted a position which allowed for hydrogen bond formation not only with Ser211, but additionally through its 3-hydroxy moiety with Asp174 (Figure 8D). Interestingly, despite the additional hydrogen bond and the occupation of the small hydrophobic pocket, the binding affinity of fragment 19 was 6-fold weaker compared to the 6-chloro-3-hydroxybenzisoxazole (17) (19, K_d_ = 15.4 ± 10.2 mM). For the 5-bromo analogue (20) the hydrophobic pocket was too small to fully accommodate the bromide substituent, and the fragment core was shifted with respect to the position observed for 16 (Figure 8A and 8C). In this position, fragment 20 was able to maintain the hydrogen bond formation with Ser211, but no further interactions with the protein were observed and, due to compound precipitation, no binding affinity was detected.

## DISCUSSION

PHGDH has recently been shown to be a potential therapeutic target in cancer types that overexpress PHGDH [4, 5]. In our study we confirmed that knockdown of PHGDH resulted in significant growth reduction in the *PHGDH*-amplified breast cancer cell line MDA-MB-468 [4]. In contrast to previous observations, however, we found that Hs578T, a breast cancer cell line with relatively high PHGDH protein expression levels, but without a genomic amplification of *PHGDH*, was insensitive to PHGDH knockdown, as was the non-amplified and low-expressing MDA-MB-231 [4]. The growth inhibition effect seen in *PHGDH*-amplified breast cancer cells, together with increasing evidence of the prevalence of PHGDH expression in certain cancer types, makes the design of a PHGDH inhibitor highly desirable to allow for further target validation using a chemical probe.

In our approach, we started from a fragment screen of a library of 600 fragments using DSF [21-23]. To validate the hits from this screen, we implemented a competition-based isothermal titration calorimetry assay. This assay allows measurement of the fragment’s binding affinity, broken down into separate thermodynamic contributions. Moreover, it tests the fragment’s ability to interfere with co-factor binding, where the DSF screen tests only the ability to bind to PHGDH. Thus, the competition ITC assay provided a relatively information-rich modality for hit validation, although at relatively low throughput. The 15 fragment hits selected from the DSF output showed a range of binding affinities in the competitive ITC experiment, confirming a non-flat SAR in ligand binding to PHGDH and providing cause for optimism for further inhibitor development.

To determine the fragment’s binding modes by X-ray crystallography, a new PHGDH construct was sought which was more stable and thus would more readily crystallise. Limited proteolysis with thermolysin revealed a stable fragment of PHGDH consisting of the core of the catalytic domain containing the cofactor-binding site, and this was shown to crystallise readily. The resultant crystals diffracted to high resolution. Comparison of crystal structures of the enzymatically active fragment sPHGDH and the shorter construct 93, showed that the cofactor-binding site was formed identically in both constructs. Using this construct, fragment hits identified by DSF and confirmed by competition ITC were soaked into PHGDH crystals. Around half of the fragments validated by ITC were shown to bind to PHGDH by X-ray crystallography, and all of these bound to the adenine-subsite. The fragment screen allowed the identification of a diverse set of new scaffolds with relatively similar K_d_ in the low mM-range. These fragments provide several avenues for further elaboration into larger fragments to increase potency and establish SARs. Through growth of fragment 16, the binding mode of some of the new modified fragments compared to the parental fragment had changed. This is likely to be due to the fact that fragment 16 initially only formed a specific interaction with one side chain (Ser211) of the protein and thus, through growth of the parental fragment, *e.g.* further addition of functional groups, the position of the new fragments can shift in order to be able to increase the number of specific interactions with the protein. In addition, the initial fragment hits are small compared to the adenine-binding site, which allows the fragments to adjust their position within the pocket.

Here, we show for the first time a fragment-based approach for the design of PHGDH binders, for which to date only two small molecule inhibitors, CBR-5884 [13] and NCT-503 [12], have been reported. The compounds identified and characterised in this work are structurally different from the reported PHGDH inhibitors CBR-5884 [13] and NCT-503 [12] (Figure 3C), and thus provide a new starting point for the design of potent and selective PHGDH inhibitors. CBR-5884 is a non-competitive inhibitor that prevents the oligomerisation of PHGDH, whereas NCT-503, also a non-competitive inhibitor in regards to substrate and cofactor, is supposed to bind close to the active site as mutation of C234 in the active site of the protein reduces the inhibitory effect of PHGDH. The presented hit fragments in this study bind competitively to NAD at the cofactor-binding site of PHGDH, and therefore serve as starting points for an orthogonal approach to PHGDH inhibition. Development of a competitive inhibitor will be valuable to investigate the effects of reversible PHGDH inhibition. In addition, studies on kinases, which represent a well-studied class of cancer targets, have shown that while drug-resistant mutants of protein kinases have emerged for both allosteric and competitive compounds, to date no resistant mutations have been reported where an allosteric and an ATP-competitive inhibitors of a given kinase have been used in combination [24, 25]. As such, it appears sensible to pursue the development of both allosteric and cofactor competitive inhibitors for oncology targets.

## MATERIALS AND METHODS

### Cell culture

Breast cancer cell lines MDA-MB-231, MDA-MB-468 and Hs578T were obtained from the American Type Culture Collection (Manassas, VA, USA). All cell lines were cultured in RPMI 1640 supplemented with 10 % FBS, penicillin (100 U/mL) and streptomycin (1.0 mg/mL) (Sigma Aldrich, St Louis, MO, USA). Lysates of other cell lines analysed for their PHGDH protein levels were obtained from research groups within the Northern Institute for Cancer Research (Newcastle University, UK).

### Transient siRNA knockdown

Transient knockdown of PHGDH was achieved in cells using a pool of four individual siRNAs (siPHGDH01 GGAAAUUGCUGUUCAGUUC, siPHG DH02 CGACAGGCUUGCUGAAUGA, siPHGDH03 GACCCUUGCUGCCGGAAAGA and siPHGDH04 UGAACUUGGUGAACGCUAA) at 5 nM (ON-TARGET plus, Dharmacon). For the non-target control, a pool of four individual siRNAs was used (UGGUUU ACAUGUCGACUAA, UGGUUUACAUGUUGUGUGA, UGGUUUACAUGUUUUCUGA, UGGUUUACAUGUUUUCCUA).

### Quantitative real-time PCR (qRT-PCR)

Total RNA was isolated using Trizol. qRT-PCR was performed in triplicate using specific primers for PHGDH (forward primer CACGACAGGCTTGCT GAATGA, reverse primer CTTCCGTAAACACGTCC AGTG) and GAPDH (forward primer CGACCACTTT GTCAAGCTCA, reverse primer GGGTCTTACT CCTTGGAGGC). qRT-PCR was performed with 0.5-0.75 μg cDNA in a final volume of 10 μL. The reactions were run in the presence of Platinum® SYBR® Green qPCR SuperMix-UDG (Thermo Fisher Scientific) and analysed through correction for GAPDH transcription level.

### Western blotting

Cells were lysed in PhosphoSafe™ extraction reagent (EMD Millipore), followed by sonication. Cell lysates (10 μg total protein/lane loaded) were separated by SDS-PAGE and transferred onto nitrocellulose membrane. Total protein content was visualised by PonceauS staining (0.1 % (wt/vol) Ponceau S in 5 % (vol/vol) acetic acid), quantified and used as loading control. Membranes were blocked in 5 % (wt/vol) milk in Tris buffered saline with 0.1 % Tween20 (TBS-T) for one hour at RT followed by incubation overnight at 4 °C with primary antibody (anti-PHGDH; Sigma Aldrich HPA021241 and anti-GAPDH; SantaCruz biotechnology Sc-25778) diluted 1:1000 in 5 % (wt/vol) milk in TBS-T. The membranes were then washed twice in TBS-T and incubated with HRP-conjugated secondary antibody diluted 1:2000 in 5 % (wt/vol) milk in TBS-T for one hour at RT. After several washes, proteins were detected by chemiluminescence using Amersham ECL Western blotting detection reagent (GE Healthcare) according to the manufacturer’s instructions. K562 cell lysate was used as a quality control against which all samples were compared.

### Colony formation assay

Following a 48 hour treatment with siRNA as described (see section transient siRNA knockdown), cells were reseeded at specific densities in complete medium. Cells were left to form colonies (> 30 cells) for up to 16 days, depending on the proliferation rate of the individual cell line. After fixation of cells with Carnoy’s fixative (methanol: acetic acid, 3:1, vol/vol), colonies were stained with 1 % crystal violet solution in 10 % aqueous ethanol. Colonies were counted by eye and cloning efficiency calculated.

### Cloning

The pNIC28-Bsa4 plasmids containing the human PHGDH cDNA encoding for the full-length enzyme (aa 1-533) as well as the truncated version (sPHGDH, aa 3-314) were kindly donated by Wyatt Yue (SGC Oxford). Both plasmids were fused with an N-terminal His_6_-tag and a Tobacco Etch Virus (TEV) protease recognition site.

### Protein expression and purification

PHGDH, sPHGDH and PHGDH-93 were expressed in *E. coli* Rosetta (DE3) grown at 37 °C in Terrific Broth (TB). Protein expression was induced with 0.5 mM isopropyl β-D-1-thiogalactopyranoside (IPTG) and the cells grown for a further 18 hours at 20 °C. Cell pellets were resuspended in extraction buffer (50 mM NaH_2_PO_4_, pH 8, 10 mM imidazole, 300 mM NaCl, 0.5 mM TCEP) supplemented with 0.05 mg/mL RNase A, 0.01 mg/mL Dnase, 0.25 mg/mL lysozyme, 5 mM MgCl_2_ and EDTA-free Complete™ protease inhibitors (Roche). Cells were lysed by sonication and then clarified by centrifugation (60,000 x g, 1 hour). PHGDH and shorter variants thereof were subsequently purified by affinity chromatography (HisTrap Ni-Sepharose column, GE Healthcare) followed by cleavage of the His_6_-tag with TEV protease (1:25 wt/wt at 4 °C overnight). Further purification of the proteins was achieved by size-exclusion chromatography (Superdex 75 26/60 (GE Healthcare)) in size exclusion buffer (25 mM HEPES, pH 7.5, 100 mM NaCl, 0.5 mM TCEP).

### Differential scanning fluorimetry

The DSF assay was performed in 384-well plates (Corning, black) in 25 mM HEPES, pH 7.5, 100 mM NaCl, 0.5 mM TCEP, 10 x SYPRO® Orange (Thermo Fisher Scientific), in a final volume of 15 μL containing 15 μM PHGDH and 2 % (vol/vol) DMSO. The fragments were added from a 100 mM master stock in 100 % DMSO to a final concentration of 1 mM using an ECHO® acoustic liquid handler (Labcyte). Reference wells contained DMSO only and 1 mM NADH was used as positive control.

The heat denaturation profile of PHGDH was followed using the fluorescence of Sypro Orange with excitation and emission wavelengths of 470 and 570 nm, respectively. Analysis was performed in GraphPad Prism using the Boltzmann equation to determine the melting temperature, T_m_, which is the inflection point of the sigmoidal denaturation curve.

### ITC competition experiments

ITC experiments were performed in 25 mM HEPES, pH 7.5, 100 mM NaCl, 0.5 mM TCEP in a MicroCal iTC200 instrument (GE Healthcare). To remove residual bound cofactor, sPHGDH was incubated with 0.5 equivalents of activated charcoal for 30 minutes at room temperature prior to performing the ITC experiments. For the competition ITC, 0.5 mM NADH mixed with the fragment of interest at 5 mM final concentration in 5 % (v/v) DMSO was titrated into 0.05 mM sPHGDH also premixed with 5 mM fragment at 25 °C. 1 x 0.5 μL, followed by 17 x 2 μL injections of ligands into protein with 120 seconds spacing between the injections was conducted. Titration of 0.5 mM NADH in 5 % DMSO into 0.05 mM sPHGDH in 5 % DMSO was used as reference. The thermodynamic parameters of the reactions were determined using ORIGIN version 7.0 (OriginLab), and the thermodynamic parameters for the fragments were derived as described [26].

### Limited proteolysis

100 μg sPHGDH or PHGDH were mixed with different proteases (papain, endoproteinase Glu-C, thermolysin, trypsin, chymotrypsin) at a molar ratio of 1:100 and 1:1000. Proteolysis was performed for one hour at RT or 37 °C prior to separation of the reaction mixture by SDS-PAGE. Characterisation of stable protein fragments was performed by liquid chromatography-mass spectrometry (LC-MS) using the liquid protein-protease mixture (Mass Spectrometry Facility, University of Leeds).

### X-ray crystallography

Crystals of PHGDH-93 were grown in 0.1 M PCTP buffer, pH 7 with 23-28 % (w/v) PEG 1500 at 20 °C by sitting drop vapour diffusion. Plates were set up using a Mosquito LCP liquid handler (TTP Labtech, Melbourn, UK) with two drops of 300 nL protein (15 mg/mL) mixed with 300 or 600 nL precipitant and a shared reservoir solution of 70 μL. Fragments were soaked into PHGDH-93 crystals at a concentration of 80 mM overnight in soaking buffer, which was a mixture of the reservoir buffer (25 % PEG 1500, 0.1 M PCTB pH 7) and the gel filtration buffer used for the protein purification (25 mM HEPES, pH 7, 100 mM NaCl, 0.5 mM TCEP) at a 3:2 ratio supplemented with 20 % (v/v) DMSO. Crystals were cryo-protected in soaking buffer supplemented with 15 % (wt/vol) PEG400 before being flash cooled in liquid nitrogen. Diffraction data were collected at the Diamond Light Source (DLS) on beamlines I02, I03, I04 and I04-1 (Supplementary Table S2). Data were processed and the structure solved using xia2 [27] and programs from the CCP4 suite [28] and the deposited structure of human PHGDH (PDB 2G76) as a starting model for molecular replacement. The structures were refined with iterative cycles of manual correction in COOT [29] followed by refinement with Refmac5 [30] within CCP4i2 [28]. Supplementary Table S2 summarises the X-ray crystallographic data collection and refinement statistics. As is often the case for low affinity ligands, the electron density suggested that multiple bound poses were present for some of the fragments for which structures were determined. The discussion and figures represent what appeared to be that pose with the highest occupancy.

## SUPPLEMENTARY MATERIALS FIGURES AND TABLES







## References

[1] Vander Heiden MG, Cantley LC, Thompson CB (2009). Understanding the Warburg effect: the metabolic requirements of cell proliferation. Science.

[2] Snell K (1984). Enzymes of serine metabolism in normal, developing and neoplastic rat tissues. Advances in enzyme regulation.

[3] Snell K, Natsumeda Y, Eble JN, Glover JL, Weber G (1988). Enzymic imbalance in serine metabolism in human colon carcinoma and rat sarcoma. British Journal of Cancer.

[4] Possemato R, Marks KM, Shaul YD, Pacold ME, Kim D, Birsoy K, Sethumadhavan S, Woo HK, Jang HG, Jha AK, Chen WW, Barrett FG, Stransky N (2011). Functional genomics reveal that the serine synthesis pathway is essential in breast cancer. Nature.

[5] Locasale JW, Grassian AR, Melman T, Lyssiotis CA, Mattaini KR, Bass AJ, Heffron G, Metallo CM, Muranen T, Sharfi H, Sasaki AT, Anastasiou D, Mullarky E (2011). Phosphoglycerate dehydrogenase diverts glycolytic flux and contributes to oncogenesis. Nature Genetics.

[6] Pollari S, Käkönen SM, Edgren H, Wolf M, Kohonen P, Sara H, Guise T, Nees M, Kallioniemi O (2011). Enhanced serine production by bone metastatic breast cancer cells stimulates osteoclastogenesis. Breast Cancer Research and Treatment.

[7] Chen J, Chung F, Yang G, Pu M, Gao H, Jiang W, Yin H, Capka V, Kasibhatla S, Laffitte B, Jaeger S, Pagliarini R, Chen Y, Zhou W (2013). Phosphoglycerate dehydrogenase is dispensable for breast tumor maintenance and growth. Oncotarget.

[8] Jing Z, Heng W, Xia L, Ning W, Yafei Q, Yao Z, Shulan Z (2015). Downregulation of phosphoglycerate dehydrogenase inhibits proliferation and enhances cisplatin sensitivity in cervical adenocarcinoma cells by regulating Bcl-2 and caspase-3. Cancer Biology & Therapy.

[9] Cho HM, Jun DY, Bae MA, Ahn JD, Kim YH (2000). Nucleotide sequence and differential expression of the human 3-phosphoglycerate dehydrogenase gene. Gene.

[10] Liu J, Guo S, Li Q, Yang L, Xia Z, Zhang L, Huang Z, Zhang N (2013). Phosphoglycerate dehydrogenase induces glioma cells proliferation and invasion by stabilizing forkhead box M1. Journal of Neuro-oncology.

[11] Jing Z, Heng W, Aiping D, Yafei Q, Shulan Z (2013). Expression and clinical significance of phosphoglycerate dehydrogenase and squamous cell carcinoma antigen in cervical cancer. International Journal of Gynecological Cancer.

[12] Pacold ME, Brimacombe KR, Chan SH, Rohde JM, Lewis CA, Swier LJ, Possemato R, Chen WW, Sullivan LB, Fiske BP, Cho S, Freinkman E, Birsoy K (2016). A PHGDH inhibitor reveals coordination of serine synthesis and one-carbon unit fate. Nature Chemical Biology.

[13] Mullarky E, Lucki NC, Beheshti Zavareh R, Anglin JL, Gomes AP, Nicolay BN, Wong JC, Christen S, Takahashi H, Singh PK, Blenis J, Warren JD, Fendt SM (2016). Identification of a small molecule inhibitor of 3-phosphoglycerate dehydrogenase to target serine biosynthesis in cancers. Proceedings of the National Academy of Sciences of the United States of America.

[14] Mullarky E, Mattaini KR, Vander Heiden MG, Cantley LC, Locasale JW (2011). PHGDH amplification and altered glucose metabolism in human melanoma. Pigment Cell & Melanoma Research.

[15] Fan J, Teng X, Liu L, Mattaini KR, Looper RE, Vander Heiden MG, Rabinowitz JD (2015). Human phosphoglycerate dehydrogenase produces the oncometabolite D-2-hydroxyglutarate. ACS Chemical Biology.

[16] Rees DC, Congreve M, Murray CW, Carr R (2004). Fragment-based lead discovery. Nature Reviews Drug Discovery.

[17] Murray CW, Verdonk ML, Rees DC (2012). Experiences in fragment-based drug discovery. Trends in Pharmacological Sciences.

[18] Congreve M, Carr R, Murray C, Jhoti H (2003). A ’rule of three’ for fragment-based lead discovery?. Drug Discovery Today.

[19] Wernimont A, Edwards A (2009). In situ proteolysis to generate crystals for structure determination: an update. PloS One.

[20] Dong A, Xu X, Edwards AM, Midwest Center for Structural Genomics, Structural Genomics Consortium (2007). In situ proteolysis for protein crystallization and structure determination. Nature Methods.

[21] Surade S, Ty N, Hengrung N, Lechartier B, Cole ST, Abell C, Blundell TL (2014). A structure-guided fragment-based approach for the discovery of allosteric inhibitors targeting the lipophilic binding site of transcription factor EthR. The Biochemical Journal.

[22] Amaning K, Lowinski M, Vallee F, Steier V, Marcireau C, Ugolini A, Delorme C, Foucalt F, McCort G, Derimay N, Andouche C, Vougier S, Llopart S (2013). The use of virtual screening and differential scanning fluorimetry for the rapid identification of fragments active against MEK1. Bioorganic & Medicinal chemistry letters.

[23] Hung AW, Silvestre HL, Wen S, Ciulli A, Blundell TL, Abell C (2009). Application of fragment growing and fragment linking to the discovery of inhibitors of Mycobacterium tuberculosis pantothenate synthetase. Angewandte Chemie.

[24] Barouch-Bentov R, Sauer K (2011). Mechanisms of drug resistance in kinases. Expert Opinion on Investigational Drugs.

[25] Rebecca VW, Smalley KS (2011). Tumor heterogeneity and strategies to overcome kinase inhibitor resistance in cancer: lessons from melanoma. Expert Opinion on Investigational Drugs.

[26] Zhang YL, Zhang ZY (1998). Low-affinity binding determined by titration calorimetry using a high-affinity coupling ligand: a thermodynamic study of ligand binding to protein tyrosine phosphatase 1B. Anal Biochem.

[27] Winter G (2010). xia2: an expert system for macromolecular crystallography data reduction. Journal of applied crystallography.

[28] Bailey S (1994). The Ccp4 Suite - Programs for Protein Crystallography. Acta Crystallogr Section D.

[29] Emsley P, Lohkamp B, Scott WG, Cowtan K (2010). Features and development of Coot. Acta Crystallographica Section D, Biological crystallography.

[30] Murshudov GN, Vagin AA, Dodson EJ (1997). Refinement of macromolecular structures by the maximum-likelihood method. Acta Crystallographica Section D, Biological crystallography.

