# Supplementary material for: Validating and enabling phosphoglycerate dehydrogenase (PHGDH) as a target for fragment-based drug discovery in PHGDH-amplified breast cancer
Source: Oncotarget. 2016 Aug 22;9(17):13139–53. doi: 10.18632/oncotarget.11487 (PMC5862567; doi:10.18632/oncotarget.11487)
Supplement: Supplementary file 1 [file oncotarget-09-13139-s001.pdf]

Validating and enabling phosphoglycerate dehydrogenase (PHGDH) as a target for fragment-based drug discovery in PHGDH-amplified breast cancer

SUPPLEMENTARY FIGURES AND TABLES

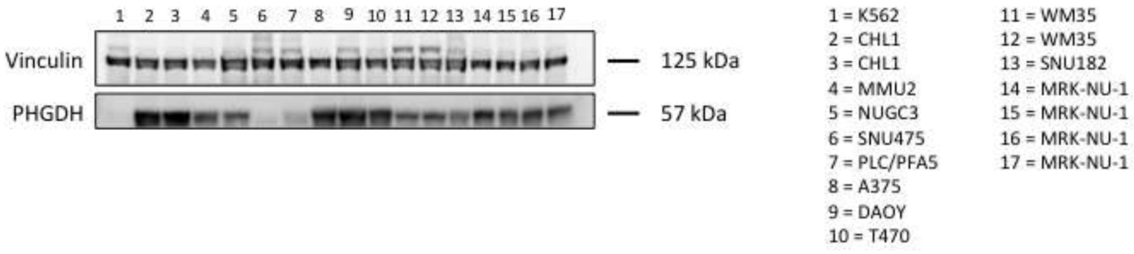

Supplementary Figure S1: Representative Western blot showing PHGDH expression in a panel of cancer cell lines. Cells were harvested, lysed and the proteins separated by gel electrophoresis. Proteins were detected by Western blotting.

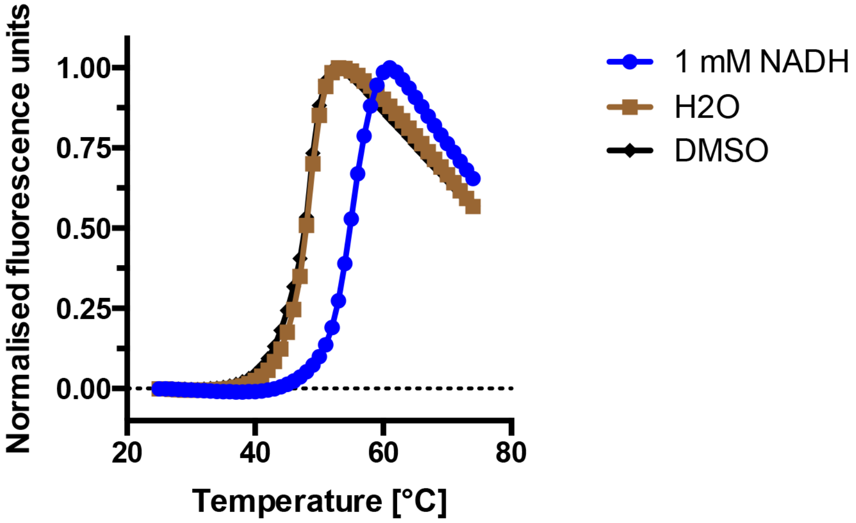

Supplementary Figure S2: Thermal profile of NADH and DMSO. Thermal denaturation profile of 15 µM PHGDH was recorded in the presence (NADH) or absence (H2O / DMSO) of 1 mM NADH. The DMSO sample contained 2 % (vol/vol) DMSO. The heat denaturation of PHGDH over 25-80 °C was followed using the fluorescence of Sypro Orange.

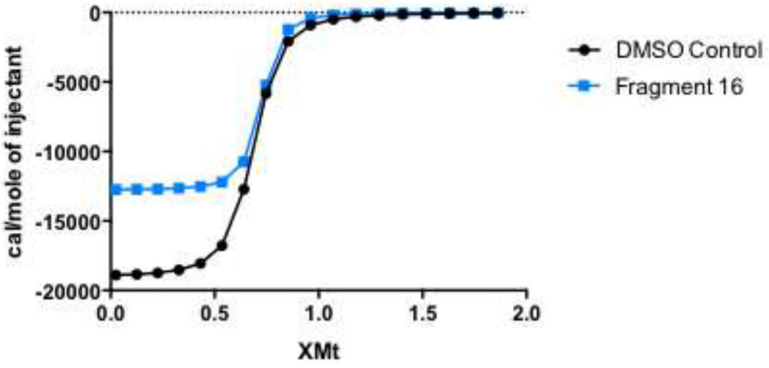

	Fragment 16
K_D (mM)	8.3 ± 2.5
ΔH (kJ mol^{-1})	-447.9
ΔS (kJ mol^{-1} K^{-1})	-1.54
ΔG (kJ mol^{-1})	11.9

Supplementary Figure S3: ITC competition experiment with fragment 16. 0.5 mM NAD^+ mixed with 5 mM fragment 16 was titrated into 0.05 mM PHGDH mixed with 5 mM fragment 16 in 25 mM HEPES, pH 7.5, 100 mM NaCl, 0.5 mM TCEP and 5 % (v/v) DMSO. Data were analysed by non-linear regression using the built-in one-site fit model of the ORIGIN software. Data showing the heat generated per mole of injected ligand against the molar ratio of receptor to ligand was plotted in GraphPad prism.

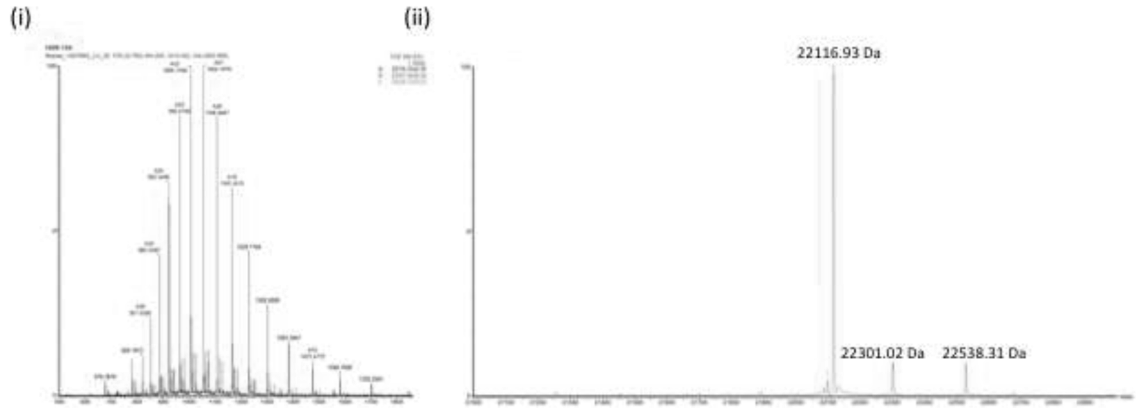

Supplementary Figure S4: Mass Spectrum analysis of fragment 93. (i) Positive ESI-MS m/z spectrum of new fragment 93, (ii) Molecular mass profile of m/z spectrum shown in (i).

Supplementary Table S1: Repurchased fragment hits from DSF screening.

See Supplementary File 1

Supplementary Table S2: Crystallographic data collection (A) and refinement statistics (B) for all new fragment-bound crystal structures from this study.

See Supplementary File 2
