# Supplementary material for: Validating and enabling phosphoglycerate dehydrogenase (PHGDH) as a target for fragment-based drug discovery in PHGDH-amplified breast cancer
Source: Oncotarget. 2016 Aug 22;9(17):13139–53. doi: 10.18632/oncotarget.11487 (PMC5862567; doi:10.18632/oncotarget.11487)
Supplement: Supplementary file 2 [file oncotarget-09-13139-s002.docx]

**Table S1: Repurchased fragment hits from DSF screening**

^a^ = average Z score of primary fragment screens by DSF;

|  | Fragment | Structure | Z score | ΔT_m_ (°C) (**screening hit**/ *repurchased fragment*) |
| --- | --- | --- | --- | --- |
| **2** | 2-Ethyl-4-methylimidazole |  | 1.0 ± 1.0 | **2.0 ± 2.1** / *-0.1 ± 0.2* |
| **3** | 1-Methyl-3-phenyl-1*H*-pyrazol-5-amine |  | 1.0 ± 0.4 | **2.2 ± 0.8** / *-0.2 ± 0.2* |
| **4** | 5-Fluoro-4-hydroxy  quinazoline |  | 1.0 ± 0.8 | **2.4 ± 1.1** / *0.2 ± 0.3* |
| **5** | 3-Chloro-4-fluorobenzamide |  | 1.0 ± 0.8 | **2.3 ± 1.0** / *-0.1 ± 0.2* |
| **6** | 7-Methyl-1*H*-indole |  | 1.1 ± 0.7 | **2.4 ± 1.2** / *0.2 ± 0.2* |
| **7** | [3-(6-Methylpyrazin-2-yl)oxyphenyl]  methanol |  | 1.2 ± 0.9 | **1.8 ± 1.7** / *-0.3 ± 0.2* |
| **8** | 6-Hydroxy-2,3-dihydrobenzo[*b*]  furan-3-one |  | 1.5 ± 0.4 | **1.7 ± 0.6** / *0.4 ± 0.4* |
| **9** | 5-Amino-1-methyl-1*H*-indole |  | 1.8 ± 1.4 | **2.0 ± 1.2** / *1.2 ± 0.4* |
| **10** | 3-(1,3-oxazol-5-yl)aniline |  | 1.0 ± 0.9 | **1.4 ± 0.6** / *-0.8 ± 0.3* |
| **11** | 3-(6-Methylpyrazin-2-yl)oxyaniline |  | 1.1 ± 0.6 | **1.7 ± 0.7** / *-0.6 ± 0.6* |
| **12** | 1-Benzylimidazole |  | 1.1 ± 0.2 | **1.3 ± 0.9** / *-0.6 ± 0.4* |
| **13** | 2,4.Dihydroxy  pyridine |  | 1.4 ± 0.2 | **0.8 ± 0.1** / *0.3 ± 0.1* |
| **14** | 5-Fluoro-2-methylbenzoic acid |  | 1.1 ± 0.2 | **1.4 ± 0.5** / *0.4 ± 0.3* |
| **15** | *N*-(3-chloro-4-methoxyphenyl)  acetamide |  | 1.2 ± 0.3 | **1.3 ± 0.2** / *-0.2 ± 0.4* |
| **16** | 3-Hydroxy  benzisoxazole |  | 1.4 ± 0.7 | **2.5 ± 0.7** / *0.2 ± 0.3* |
