# Supplementary material for: Validating and enabling phosphoglycerate dehydrogenase (PHGDH) as a target for fragment-based drug discovery in PHGDH-amplified breast cancer
Source: Oncotarget. 2016 Aug 22;9(17):13139–53. doi: 10.18632/oncotarget.11487 (PMC5862567; doi:10.18632/oncotarget.11487)
Supplement: Supplementary file 3 [file oncotarget-09-13139-s003.docx]

**Table S2: Crystallographic data collection (A) and refinement statistics (B) for all new fragment-bound crystal structures from this study.**

**(A) Data collection statistics**

|  | **PHGDH-93 with NAD^+^** | **Fragment 3** | **Fragment 5** | **Fragment 9** | **Fragment 10** | **Fragment 14** | **Fragment 15** | **Fragment 16** |
| --- | --- | --- | --- | --- | --- | --- | --- | --- |
| **Cell dimensions** |  |  |  |  |  |  |  |  |
| **a, b, c (Å)** | 43.4, 50.3, 53.2 | 43.4, 45.8, 54.8 | 43.4, 45.8, 56.0 | 43.3, 45.8, 56.1 | 43.3, 45.9, 56.2 | 43.3, 45.6, 56.3 | 43.4, 45.4, 55.3 | 43.4, 45.6, 55.4 |
| **α, β, γ (°)** | 98.6, 112.6, 104.3 | 97.0, 110.1, 106.7 | 97.6, 111.0, 106.0 | 97.9, 110.7, 106.4 | 97.9, 111.0, 106.0 | 98.0, 111.1, 105.9 | 97.9, 110.1, 106.3 | 97.2, 110.5, 106.7 |
| **Resolution range (Higher shell)** | 47.2 – 1.3 (1.32-1.30) | 49.9 - 1.3 (1.33-1.31) | 50.5 - 1.5 (1.53-1.5) | 50.6 - 1.5 (1.53-1.5) | 50.6 - 1.5 (1.53-1.50) | 50.6 - 1.5 (1.53-1.5) | 50.1 - 1.5 (1.53-1.50) | 50.2 - 1.3 (1.33-1.31) |
| **Observations** | 162265 (7837) | 143356 (7246) | 111440 (5460) | 108505 (5089) | 110551 (5562) | 104812 (4912) | 109521 (5414) | 147779 (7337) |
| **Unique reflections** | 82885 (4003) | 73976 (3742) | 51690 (2562) | 51430 (2547) | 51377 (2564) | 54400 (2659) | 51238 (2530) | 75610 (3737) |
| **I/σ(I)** | 6.1 (0.8) | 4.0 (0.7) | 5.9 (1.7) | 6.3 (2.8) | 4.7 (0.9) | 5.0 (1.2) | 4.6 (0.9) | 4.5 (1.0) |
| **Rmerge (all I+ and I-)** | 0.059 (0.987) | 0.091 (0.917) | 0.056 (0.417) | 0.064 (0.376) | 0.094 (0.564) | 0.084 (1.241) | 0.111 (1.088) | 0.058 (0.622) |
| **Rpim (all I+ & I-)** | 0.059 (0.987) | 0.091 (0.917) | 0.050 (0.383) | 0.058 (0.344) | 0.087 (0.519) | 0.076 (1.141) | 0.1 (0.972) | 0.058 (0.622) |
| **Half-set correlation CC(1/2)** | 0.992 (0.416) | 0.987 (0.352) | 0.98 (0.645) | 0.99 (0.804) | 0.986 (0.585) | 0.977 (0.598) | 0.989 (0.355) | 0.996 (0.563) |

|  | **Fragment 17** | **Fragment 18** | **Fragment 19** | **Fragment 20** |
| --- | --- | --- | --- | --- |
| **Cell dimensions** |  |  |  |  |
| **a, b, c (Å)** | 43.2, 45.5, 55.7, | 43.4, 45.8, 56.1 | 43.3, 45.6, 56.2 | 43.3, 45.5, 55.8 |
| **α, β, γ (°)** | 97.7, 111.1, 105.8 | 97.6, 111.1, 106.2 | 97.9, 111.1, 106.0 | 97.6, 111.1, 105.9 |
| **Resolution range (Higher shell)** | 50.3 - 1.6  (1.61-1.58) | 49.9 - 1.6 (1.60-1.57) | 50.6-1.2 (1.50-1.47) | 50.3 - 1.4 (1.37-1.35) |
| **Observations** | 85616 (2939) | 93665 (4464) | 118832 (5212) | 150732 (5614) |
| **Unique reflections** | 45552 (1779) | 48549 (2420) | 60640 (2896) | 76482 (2975) |
| **I/σ(I)** | 7.5 (1.7) | 4.4 (1.6) | 7.8 (1.6) | 5.3 (1.2) |
| **Rmerge (all I+ and I-)** | 0.051 (0.267) | 0.089 (0.210) | 0.039 (0.348) | 0.057 (0.641) |
| **Rpim (all I+ & I-)** | 0.047 (0.248) | 0.081 (0.194) | 0.036 (0.321) | 0.050 (0.579) |
| **Half-set correlation CC(1/2)** | 0.993 (0.887) | 0.974 (0.932) | 0.996 (0.911) | 0.994 (0.363) |

**(B) Refinement statistics**

|  | **PHGDH-93 with NAD^+^** | **Fragment 3** | **Fragment 5** | **Fragment 9** | **Fragment 10** | **Fragment 14** | **Fragment 15** | **Fragment 16** |
| --- | --- | --- | --- | --- | --- | --- | --- | --- |
| **No. reflections all/free** | 82884 / 4162 | 73958 / 3651 | 51644 / 2599 | 51276 / 2653 | 51257 / 2653 | 54097 / 2775 | 51238 / 2651 | 75604 / 3878 |
| **R-factor/R-free** | 0.16 / 0.20 | 0.19 / 0.24 | 0.18 / 0.24 | 0.19 / 0.25 | 0.23 / 0.29 | 0.22 / 0.27 | 0.19 / 0.24 | 0.18 / 0.22 |
| **RMS Deviations (Bonds)** | 0.0270 | 0.0186 | 0.0219 | 0.0218 | 0.0217 | 0.0248 | 0.0183 | 0.0203 |
| **RMS Deviations (Angles)** | 2.387 | 1.962 | 2.067 | 2.155 | 2.051 | 2.296 | 1.978 | 2.069 |
| **Mean Bfactor (count)** |  |  |  |  |  |  |  |  |
| **Protein** | 52.2 (6178) | 39.7 (6154) | 36.3 (6160) | 30.7 (6170) | 43.1 (6122) | 37.9 (6170) | 38.7 (6154) | 36.1 (6141) |
| **Ligand** | 45.2 (132) | 60.4 (48) | 60.8 (32) | 37.8 (42) | 68.6 (40) | 46.5 (34) | 70.3 (46) | 68.4 (30) |
| **Solvent** | 37.4 (245) | 32.5 (272) | 27.1 (210) | 26.9 (305) | 35.7 (264) | 29.6 (275) | 31.2 (238) | 22.7 (167) |

|  | **Fragment 17** | **Fragment 18** | **Fragment 19** | **Fragment 20** |
| --- | --- | --- | --- | --- |
| No. reflections all/free | 45538 /2245 | 44285/ 2035 | 80909 / 2993 | 76482 / 3718 |
| R-factor/R-free | 0.17 / 0.22 | 0.19 / 0.24 | 0.22 / 0.28 | 0.18 / 0.22 |
| RMS Deviations (Bonds) | 0.0181 | 0.0218 | 0.04 | 0.0283 |
| RMS Deviations (Angles) | 1.887 | 2.030 | 2.769 | 2.487 |
| Mean Bfactor (count) |  |  |  |  |
| Protein | 39.7 (6154) | 30.9 (6154) | 44.1 (6128) | 44.8 (6059) |
| Ligand | 75.4 (56) | 48.0 (36) | 54.1 (28) | 101.1 (28) |
| Solvent | 24.5 (186) | 18.0 (133) | 19.5 (111) | 25.3 (133) |
